# Knockdown of lncRNA LINC01234 Suppresses the Tumorigenesis of Liver Cancer *via* Sponging miR-513a-5p

**DOI:** 10.3389/fonc.2020.571565

**Published:** 2020-10-16

**Authors:** Wen Xu, Kesang Li, Changfeng Song, Xiaotong Wang, Yueqi Li, Baixue Xu, Xin Liang, Wanli Deng, Junqing Wang, Jianwen Liu

**Affiliations:** ^1^State Key Laboratory of Bioreactor Engineering and Shanghai Key Laboratory of New Drug Design, School of Pharmacy, East China University of Science and Technology, Shanghai, China; ^2^Department of Hematology and Oncology, Hwa Mei Hospital, Ningbo Institute of Life and Health Industry, University of Chinese Academy of Sciences, Ningbo, China; ^3^Key Laboratory of Diagnosis and Treatment of Digestive System Tumors of Zhejiang Province, Ningbo, China; ^4^Department of TCM Oncology, Putuo District Central Hospital, Shanghai University of Traditional Chinese Medicine, Shanghai, China; ^5^Department of Hepatobiliary Surgery, Ruijin Hospital, Shanghai Jiao Tong University School of Medicine, Shanghai, China

**Keywords:** liver cancer, LINC01234, USP4, miR-513a-5p, TGF-β

## Abstract

**Background:**

Liver cancer is a frequent malignancy with poor prognosis and high mortality all over the world. It has been reported many lncRNAs could modulate the tumorigenesis of liver cancer. To identify novel potential targets for liver cancer, the differential expressed lncRNAs between liver cancer and adjacent normal tissues was analyzed with bioinformatics tool.

**Methods:**

The differential expressed lncRNAs between liver cancer and adjacent normal tissues were analyzed with bioinformatics tool. Cell viability and proliferation was tested by CCK8 and Ki67, respectively. Apoptosis of liver cancer cells was tested by flow cytometry. Gene and protein expressions in liver cancer cells were measured by qRT-PCR and western blot, respectively. *In vivo* model of liver cancer was established to detect the effect of LINC01234 on liver cancer *in vivo*.

**Results:**

LINC01234 was found to be negatively correlated with the survival rate of patients with liver cancer. Moreover, knockdown of LINC01234 significantly suppressed the proliferation and invasion of liver cancer cells via inducing the apoptosis. Meanwhile, miR-513a-5p was sponged by LINC01234, and USP4 was found to be a direct target of miR-513a-5p. In addition, LINC01234 knockdown inhibited the tumorigenesis of liver cancer via inactivating TGF-β signaling. Furthermore, silencing of LINC01234 notably inhibited the tumor growth of liver cancer *in vivo*.

**Conclusion:**

Downregulation of LINC01234 could inhibit the tumorigenesis of liver cancer via mediation of miR-513a-5p/USP4/TGF-β axis. Thus, LINC01234 might serve as a new target for the treatment of liver cancer.

## Background

Liver cancer is a common malignant tumor, with a 15–17% 5-year survival rate (1, 2). The prognosis of patients with liver cancer is poor because of the high incidence of postoperative recurrence and metastasis (3). Surgery is still the main treatment strategy; however, liver cancer patients are usually diagnosed at advanced stages. Thus, they often miss the optimal opportunity for surgical resection (4). Furthermore, liver cancer is highly resistant to conventional chemotherapy and radiation therapy (5, 6). Currently, clinicopathologic prognostic factors include TNM stage, tumor size, tumor rupture and underlying cirrhosis (7). In addition to these traditional clinical prognostic factors, genetic biomarkers are novel indicators of liver cancer diagnosis and prognosis (8). Molecular biomarkers could predict patient prognosis and served as the target for the treatment of liver cancer (9), while it is still a lack of therapeutic markers for liver cancer. Therefore, it is necessary to adopt a comprehensive approach to identify novel biomarkers for the treatment of liver cancer.

LncRNAs are a class of non-coding RNA transcripts with the length of about 200 nucleotides (10). Intracellular lncRNAs are known to suppress the biological activity of miRNAs and regulate the expression of mRNAs targeted by miRNAs through competing endogenous RNAs (ceRNAs) (11). Therefore, lncRNAs have been considered to be important targets for treating multiple diseases (12, 13). Moreover, lncRNAs play key roles in malignant tumors, including liver cancer (14, 15). However, the effect of lncRNAs on tumorigenesis of liver cancer remains to be further explored.

MicroRNAs (miRNAs) are a class of non-coding small ribonucleic acids which can regulate gene expression by suppression of mRNA translation or degradation of mRNAs (16). Moreover, miRNAs have been considered as important biological regulators for multiple diseases, including liver cancer (1, 17). Meanwhile, in previous studies, lncRNAs have been reported to mediate the tumorigenesis of liver cancer via sponging miRNAs (15, 18). For example, Hong et al. (19) found that lncRNA LINC00460 can contribute to tumor growth and metastasis of hepatocellular carcinoma through miR-342-3p-dependent AGR2 up-regulation. Thus, miRNAs play a key role in liver cancer.

In the current study, we aimed to detect the differentially expressed lncRNAs, which is closely associated with the tumorigenesis of liver cancer. We hope the research could provide a new idea for the development of novel therapeutic strategies against liver cancer.

## Materials and Methods

### Cell Culture

Liver cancer cell lines (HepG2 and Huh-7), normal liver cell lines (L-02) and 293T cells were purchased from the American Type Culture Collection (ATCC, Manassas, VA, United States) and cultured in Dulbecco’s Modified Eagle’s Medium (DMEM, Thermo Fisher Scientific, Waltham, MA, United States), supplemented with 10% FBS, 1% penicillin and streptomycin (Thermo Fisher Scientific) in an incubator (37°C, 5% CO_2_).

### Bioinformatics Analysis

The gene expression data of liver cancer and adjacent normal tissue was obtained from dataset (GSE113850) and the Cancer Genome Atlas (TCGA). The survival curve and the correlation between tumor stage of liver cancer were calculated based on the data from TCGA.

### Quantitative Real Time Polymerase Chain Reaction

Total RNA was extracted from liver cancer cell lines using TRIzol reagent (TaKaRa, Tokyo, Japan) according to the manufacturer’s protocol. First-strand cDNA was synthesized using the PrimeScript RT reagent Kit (Takara) according to the manufacturer’s protocol. qRT-PCR was performed in an ABI7500 system using SYBR Green methods. qRT-PCR was performed in triplicate with the following protocol: 2 min at 94°C, followed by 35 cycles (30 s at 94°C and 45 s at 55°C). The primers for all the genes were obtained from GenePharma (Shanghai, China). 2^–ΔΔCT^ method was used to quantify the data. The internal reference gene (U6 or β-actin) was used for normalization. The primers were obtained from GenePharma (Shanghai, China). LINC01234: forward, 5′-TCTCACCTTTCAAACGCTTGTC-3′ and reverse 5′-ACTCTCCTTCCTTTCCTCTGATTC-3′. MiR- 513a-5p: forward, 5′-AGGGAGGTGTCATCTCAACTGA-3′ and reverse 5′-CTCAACTGGTGTCGTGGAGTC-3′. β-actin: forward, 5′-GTCCACCGCAAATGCTTCTA-3′ and reverse 5′-TGCTGTCACCTTCACCGTTC-3′. USP4: forward, 5′-GGA CTATGTATTGGTCCCTACCG-3′ and reverse 5′-TCGATGG TTGCAATGGTGTC-3′. U6: forward, 5′-CTCGCTTCGGCAG CACAT-3′ and reverse 5′-AACGCTTCACGAATTTGCGT-3′.

### Cell Transfection

Lentiviral expressing short-hairpin RNA (shRNA1 or shRNA2) directed target LINC01234 and one non-targeting sequence (negative control) were obtained from Hanbio Biotechnology Co., Ltd (Shanghai, China). Next, LINC01234 shRNA1 or shRNA2 was packaged into lentiviruses. Then the lentiviral vector DNAs were then transfected into 293T cells including lenti-LINC01234 shRNAs and negative control (NC). After transfection, the cells were incubated at 37°C, and then the supernatant was collected. After that, supernatants of two LINC01234 shRNAs and negative control were filtered into particles. Finally, all liver cancer cells were infected with lentiviral particles according to the manufactures’ protocol. After 48 h of incubation, stable liver cancer cells were then selected by puromycin (2.5 μg/mL, Sigma Aldrich, St. Louis, MO, United States). Green fluorescence and qRT-PCR were used to verify the efficiency of transfection.

For miR-513a-5p transfection, liver cancer cells were transfected with miR-513a-5p agomir, miR-513a-5p antagomir or NC by Lipofectamine 2000 according to the previous reference (20). MiR-513a-5p agomir, miR-513a-5p antagomir and negative control RNAs were purchased from GenePharma (Shanghai, China). The efficiency of transfection was detected by q-PCR.

### CCK-8 Assay

Liver cancer cells were seeded in 96-well plates (5 × 10^3^ per well) overnight. Then, cells were treated with negative control (NC) or LINC01234 shRNA1 for 0, 24, 48 and 72 h, respectively. Subsequently, cells were treated with 10 μl CCK-8 reagents (Beyotime, Shanghai, China) and further incubated for 2 h at 37°C. Finally, the absorbance of liver cancer cells was measured at 450 nm using a microplate reader (Thermo Fisher Scientific).

### Cell Apoptosis Analysis

Liver cancer cells were trypsinized, washed with phosphate buffered saline and resuspended in Annexin V Binding Buffer. Then, cells were stained with 5 μl FITC and 5 μl propidium (PI) in the dark for 15 min. Cells were analyzed using flow cytometer (BD, Franklin Lake, NJ, United States) to test the cell apoptosis rate.

### Cell Invasion Assay

For cell invasion analysis, transwell assay was performed in this study. The upper chamber is pre-treated with 100 μl of Matrigel. Huh7 cells were seeded into the upper chamber in media with 1% FBS, and the density was adjusted to about 1.0 × 10^6^ cells per chamber. RPMI1640 medium with 10% FBS was added in the lower chamber. After 24 h of incubation at 37°C, the transwell chamber was rinsed twice with PBS (5 min per time), fixed by 5% glutaraldehyde at 4°C and stained with 0.1% crystal violet for 30 minutes. The transwell chamber was washed twice with PBS and then observed under a microscope. The number of invaded cells was regarded to be a reflection of the invasion ability.

### Dual Luciferase Reporter Assay

The partial sequences of LINC01234 and 3′-UTR of USP4 containing the putative binding sites of miR-513a-5p were synthetized and obtained from Sangon Biotech (Shanghai, China), then were cloned into the pmirGLO Dual-Luciferase miRNA Target Expression Vectors (Promega, Madison, WI, United States) to construct wild-type reporter vectors LINC01234 (WT/MT) and USP4 (WT/MT), respectively. The LINC01234 (WT/MT) or USP4 (WT/MT) were transfected into 293T cells together with control, vector-control (NC) or miR-513a-5p agomir using Lipofectamine 2000 (Thermo Fisher Scientific) according to the manufacturer’s instructions. The relative luciferase activity was analyzed by the Dual-Glo Luciferase Assay System (Promega). The relative luciferase activity was analyzed by the Dual-Glo Luciferase Assay System (Promega).

### RNA Pull-Down

For the RNA pulldown assay, the Biotin RNA Labeling Mix (Roche, Basel, Switzerland) was used to transcribe and label probe-control or probe-LINC01234 from LINC01234 shRNA lenti vector *in vitro*. An RNA structure buffer (Thermo, MA, United States) was used to induce secondary structure formation from the biotin-labeled RNAs. The biotinylated LINC01234 and negative control (bio-NC) were generated via GenePharma and coated to streptavidin-conjugated magnetic beads. Liver cancer cells were lysed and then incubated with the magnetic beads for 6 h. The RNA on the beads was isolated and the enrichment level of miR-513a-5p was detected by PCR.

### Wound Healing Assay

Huh-7 cells were plated into a 24-well Cell Culture Cluster and allowed to grow to 80–90% confluence. Then, cells were underlined perpendicular to the cell culture plate with a small pipette head. After washing with PBS 3 times, serum-free medium was used for further culture, and the scratch widths at 0 and 48 h were recorded under an optical microscope.

### Immunofluorescence

Liver cancer cells or tumor tissues of mice were prefixed in 4% paraform for 10 min, and fixed in pre-cold methanol for another 10 min. Next, cells were incubated with primary antibodies overnight at 4(C: anti-Ki67 (Abcam; 1:1000), anti-Smad4 (Abcam; 1:1000). Goat anti-rabbit IgG antibody (Abcam; 1:5000) was used as the secondary antibody. The nuclei were stained with DAPI for 15 min at room temperature. The samples were visualized by fluorescence microscope (Olympus CX23, Tokyo, Japan) immediately.

### Western Blot Detection

Total protein was isolated from cell lysates or tissues by using RIPA buffer. The concentration of protein was detected with a BCA protein kit (Thermo Fisher Scientific). Then, proteins (40 μg per lane) were separated with 10% SDS-PAGE gel and then transferred into polyvinylidene fluoride (PVDF, Thermo Fisher Scientific) membranes. After blocking with 3% skim milk for 1 h, the membranes were incubated with primary antibodies at 4°C overnight. Subsequently, membranes were incubated with secondary anti-rabbit antibody (Abcam; 1:5000) at room temperature for 1 h. Membranes were scanned by using an Odyssey Imaging System and analyzed with Odyssey v2.0 software (LICOR Biosciences, Lincoln, NE, United States). The primary antibodies used in this study as follows: anti-E-cadherin (Abcam, Cambridge, MA, United States; 1:1000), anti-vimentin (Abcam; 1:1000), anti-α-SMA (Abcam; 1:1000), anti-smad2 (Abcam; 1:1000), anti-smad3 (Abcam; 1:1000), anti-USP4 (Abcam; 1:1000), anti-cleaved caspase 3 (Abcam; 1:1000), anti-Akt (Abcam; 1:1000), anti-ERK (Abcam; 1:1000) and anti-β-actin (Abcam; 1:1000). β-actin was used as an internal control.

### *In vivo* Study

18 BALB/c nude mice (6–8 weeks old) were purchased from Vital River (Beijing, China). The mice were housed within a dedicated SPF facility. Huh7 stable expressed LINC01234 shRNA1 cells were transplanted subcutaneously in each mouse according to the previous reference (21). The tumor volume was measured weekly according to the reference (22). At the end of the experiments, mice were sacrificed and the tumors were collected and weighted. The expression of p-smad2 and p-smad3 were detected by immunohistochemistry (IHC) staining as previously reported (19). All *in vivo* experiments were performed in accordance with National Institutes of Health guide for the care and use of laboratory animals, following a protocol approved by the Ethics Committees of East China University of Science and Technology.

### TUNEL Staining

Apoptosis was also determined by the TUNEL assay according to the manufacturer’s instructions. Briefly, paraffin sections were washed, permeabilized, and then incubated with 50 μl TUNEL reaction mixtures in a wet box for 60 min at 37°C in the dark. For signal conversion, slides were incubated with 50 μl of peroxidase (POD) for 30 min at 37°C, rinsed with PBS, and then incubated with 50 μl diaminobenzidine (DAB) substrate solution for 10 min at 25°C. Finally, the expression of apoptotic cells was observed under an optical microscope.

### Statistical Analysis

Each group were performed three independent experiments and all data were expressed as the mean ± standard deviation (SD). Differences were analyzed using Student’s *t*-test (only 2 groups) or one-way analysis of variance (ANOVA) followed by Tukey’s test (more than 2 groups, Graphpad Prism7). *P* < 0.05 was considered to indicate a statistically significant difference.

## Results

### Differentially Expressed lncRNAs Between Liver Cancer and Adjacent Normal Tissues

**FIGURE 1:**
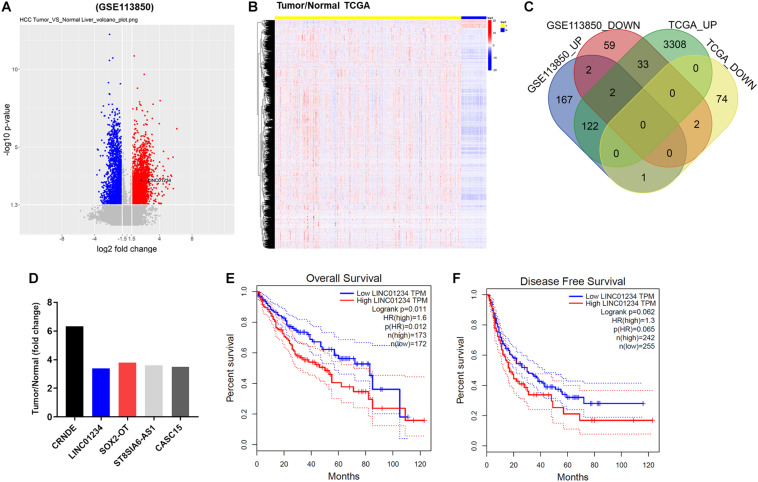
The differentially expressed lncRNAs between liver cancer and adjacent normal tissues. **(A)** Volcano plots illustrating the lncRNAs differentially expressed in liver cancer detected in the GSE113850 dataset. Red indicates a higher expression level, while blue indicates a lower expression level. **(B)** Differentially expressed lncRNAs among normal and liver cancer tissues were presented by TCGA. **(C)** Venn diagram showing the overlap among the differentially expressed lncRNAs in the GSE113850 dataset. **(D)** Dataset indicated overlap 5 upregulated lncRNAs in liver cancer tissues. **(E,F)** Overall and disease free survival of liver cancer were analyzed by TCGA.

To detect differentially expressed lncRNAs between liver cancer and normal tissues, a bioinformatics analysis as performed. As indicated in Figure 1A, differentially expressed lncRNAs in GSE113850 data set (DS) were presented using Volcano Plot. In addition, differentially expressed lncRNAs among normal and tumor tissues were also presented in TCGA (Figure 1B). Among these differentially expressed lncRNAs in GSE113850 DS and TCGA, 59 were commonly downregulated, whereas 167 were commonly upregulated (Figure 1C). Moreover, 5 most upregulated overlap linRNAs in liver cancer were presented (Figure 1D). Among these 5 lncRNAs, high expression of LINC01234 was negatively correlated with the survival rate of patient with liver cancer (Figures 1E,F). These data suggested that LINC01234 might play an important role during the tumorigenesis of liver cancer. Thus, LINC01234 was selected for investigation in the following experiments.

### LINC01234 Knockdown Significantly Decreased the Proliferation of Liver Cancer Cells

**FIGURE 2:**
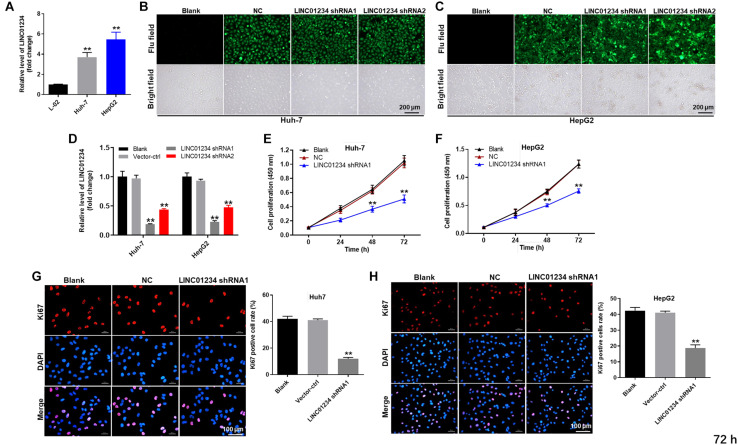
LINC01234 knockdown significantly decreased the proliferation of liver cancer cells. **(A)** The expression of LINC01234 in Huh-7, HepG2 and L-02 cells was detected by qRT-PCR. **(B,C)** Huh-7 cells were transfected with LINC01234 shRNA1 or shRNA2 for 24 h. Then, the efficiency of cell transfection was detected by green fluorescence staining. **(D)** The expression of LINC01234 in liver cancer cells was detected by qRT-PCR. The proliferation of **(E)** Huh-7 or **(F)** HepG2 cells was detected by CCK-8 assay. **(G,H)** Ki67 staining was performed to test the proliferation of Huh-7 and HepG2 cells. Red indicates the Ki67 staining. Blue indicates DAPI staining. **P < 0.01 compared to control.

In order to test the gene expression, qRT-PCR was used. As indicated in Figure 2A, the expression of LINC01234 in liver cancer cells was much higher than that in normal liver cells. Moreover, LINC01234 shRNA1 and shRNA2 were stably transfected into liver cancer cells (Figures 2B,C), and the expression of LINC01234 in liver cancers was significantly downregulated when transfected with LINC01234 shRNA (Figure 2D). Since LINC01234 shRNA1 exhibited better transfection efficiency, it was therefore used in subsequent experiments. Furthermore, the viability and proliferation of Huh-7 and HepG2 cells were significantly inhibited by LINC01234 knockdown (Figures 2E–H). Meanwhile, Huh-7 cells were more sensitive to LINC01234 shRNA1 than HepG2 cells. Thus, Huh-7 cells were used in the following experiments. Taken together, LINC01234 silencing significantly decreased the proliferation of liver cancer cells.

### LINC01234 Sponged miR-513a-5p

**FIGURE 3:**
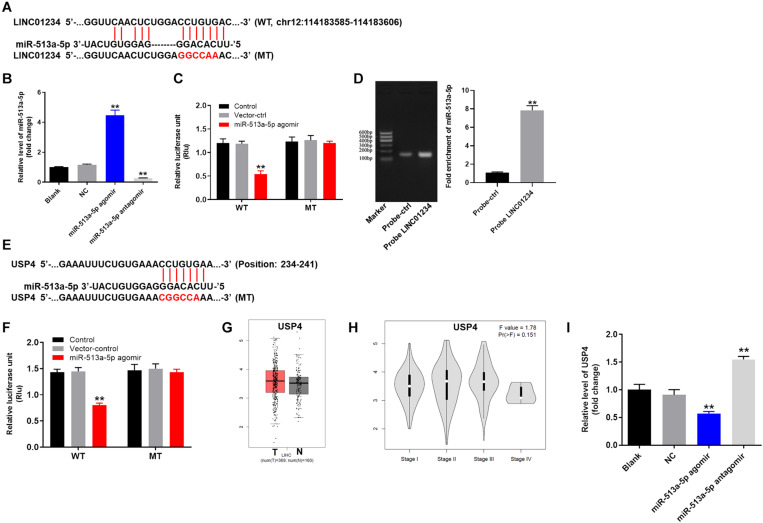
LINC01234 sponged miR-513a-5p. **(A)** Gene structure of LINC01234 indicating the predicted miR-513a-5p binding site in its 3′UTR. **(B)** Huh-7 cells were transfected with miR-513a-5p agomir/antagomir for 24 h. Then, cell transfection was verified by qRT-PCR. **(C)** The luciferase activity in Huh-7 cells after co-transfecting a plasmid encoding the wild-type (WT) or mutant (MT) LINC01234 3′-UTR and miR-513a-5p. **(D)** Co-localization of LINC01234 and miR-513a-5p detected using RNA pull-down. **(E)** Gene structure of USP4 indicating the predicted miR-513a-5p binding site in its 3′UTR. **(F)** The luciferase activity in Huh-7 cells after co-transfecting a plasmid encoding the wild-type (WT) or mutant (MT) USP4 3′-UTR and miR-513a-5p. **(G)** The differential expression of USP4 in normal and tumor tissues was analyzed from the data of TCGA. **(H)** The correlation between USP4 and different stages of liver cancer was analyzed. **(I)** The expression of USP4 in liver cancer cells was measured by qRT-PCR. **P < 0.01 compared to control.

For the purpose of exploring the mechanism by which LINC01234 mediated the progression of liver cancer, Starbase^1^ was performed. As shown in Figure 3A, LINC01234 had a putative miR-513a-5p targeting site. In addition, miR-513a-5p agomir/antagomir was stably transfected into Huh-7 cells (Figure 3B). Meanwhile, co-transfection of the wild-type LINC01234 vector (WT-LINC01234) with miR-513a-5p agomir, significantly reduced luciferase activities compared with mutant LINC01234 vector (MT-LINC01234) (Figure 3C). Additionally, LINC01234 bound to miR-513a-5p (Figure 3D) was verified with pull down assay. Moreover, USP4 was found to be the direct target of miR-513a-5p (Figures 3E,F). However, the difference of USP4 level between liver cancer and adjacent normal tissues is not significant (Figure 3G). Besides, the difference of USP4 expression among different stages of liver cancer is not obvious (Figure 3H). Additionally, the expression of USP4 in liver cancer cells was notably upregulated by miR-513a-5p antagomir but inhibited by miR-513a-5p agomir (Figure 3I). Altogether, LINC01234 knockdown inhibited the tumorigenesis of liver cancer via mediation of miR-513a-5p/USP4 axis.

### Knockdown of LINC01234 Significantly Suppressed the Progression of Liver Cancer *in vitro*

**FIGURE 4:**
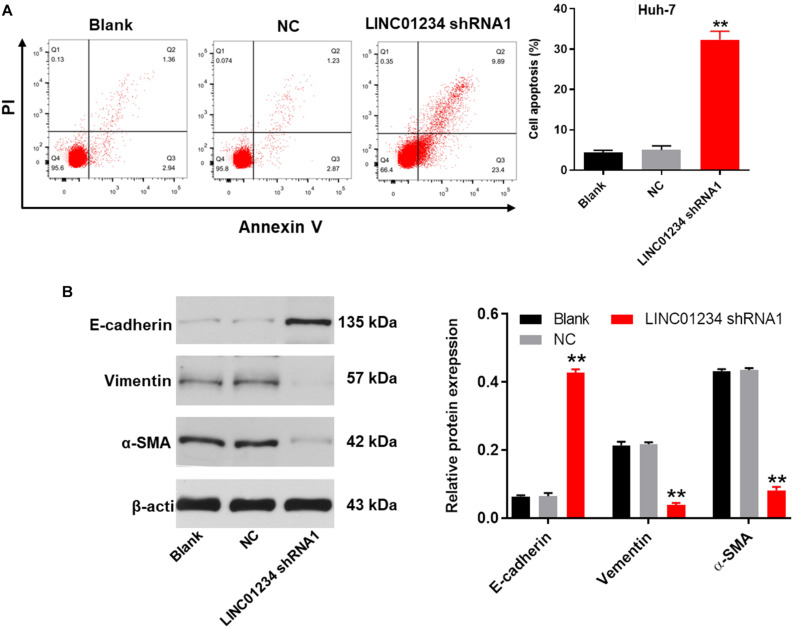
Knockdown of LINC01234 significantly suppressed the progression of liver cancer *in vitro*. **(A)** The apoptotic Huh-7 cells were examined by flow cytometry. **(B)** Cell migration was measured by wound healing assay. **(C)** Cell invasion was detected by transwell assay. **(D)** The protein expressions of E-cadherin, vimentin and α-SMA in Huh-7 cells were detected by western blot. The relative protein expressions were quantified by normalizing to β-actin. **P < 0.01 compared to control.

Next, flow cytometry was performed to detect the cell apoptosis. As revealed in Figure 4A and Supplementary Figure S1, downregulation of LINC01234 notably induced the apoptosis of liver cancer cells. In addition, knockdown of LINC01234 greatly inhibited the migration and invasion of liver cancer cells (Figures 4B,C). Furthermore, the expression of E-cadherin in Huh-7 cells was significantly upregulated by LINC01234 shRNA1 (Figure 4D). In contrast, knockdown of LINC01234 greatly decreased the expression of vimentin and α-SMA in liver cancer cells (Figure 4D). To sum up, knockdown of LINC01234 significantly suppressed the progression of liver cancer *in vitro*.

### Downregulation of LINC01234 Notably Inhibited the Tumorigenesis of Liver Cancer via Mediation of miR-513a-5p/USP4/TGF-β1 Axis

**FIGURE 5:**
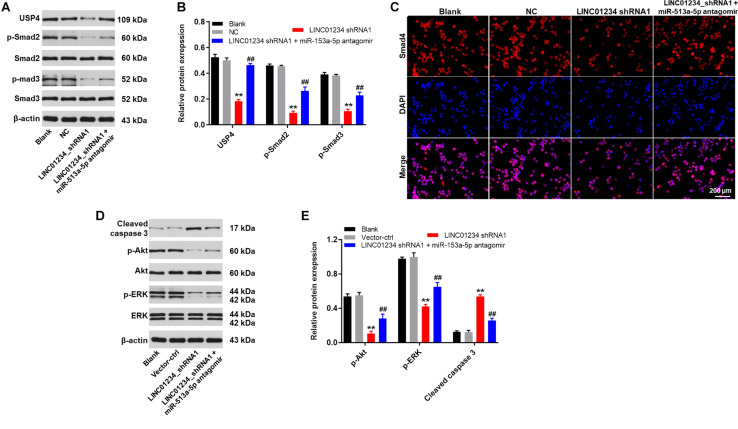
Downregulation of LINC01234 notably inhibited the tumorigenesis of liver cancer via mediation of miR-513a-5p/USP4/TGF-β1 axis. **(A)** The protein expressions of USP4, Smad2, Smad3, p-Smad2 and p-Smad3 in Huh-7 cells were detected by western blot. **(B)** The relative protein expressions were quantified by normalizing to β-actin. **(C)** The location of Smad4 was explored by immunofluorescence staining. Red indicates Smad4 fluorescence. Blue indicates DAPI fluorescence. **(D)** The protein expressions of Akt, p-Akt, cleaved caspase 3, ERK and p-ERK in Huh-7 cells were measured by western blot. **(E)** The relative protein expressions were quantified by normalizing to β-actin. **P < 0.01 compared to control. ^##^*P* < 0.01 compared to LINC01234 shRNA1.

In order to investigate the protein expressions in liver cancer cells, western blot was used. As indicated in Figures 5A,B, the expressions of USP4, p-Smad2 and p-Smad3 in Huh-7 cells were significantly downregulated in the presence of LINC01234 shRNA1, while this phenomenon was partially reversed by miR-513a-5p antagomir. In addition, the expression of Smad4 in cytoplasm of Huh-7 cells was significantly decreased by LINC01234 knockdown, which was partially reversed in the presence of miR-513a-5p antagomir (Figure 5C). This data suggested that Smad4 transferred from the cytoplasm to the nucleus in the presence of LINC01234 shRNA1. Moreover, LINC01234 shRNA1 greatly suppressed the expressions of p-Akt and p-ERK in Huh-7 cells. In contrast, the expression of cleaved caspase 3 in Huh-7 cells was notably upregulated in the presence of LINC01234 knockdown (Figures 5D,E). However, downregulation of miR-513a-5p partially reversed the effect of LINC01234 shRNA1 on these proteins (Figures 5D,E). In summary, silencing of LINC01234 notably inhibited the tumorigenesis of liver cancer via mediation of miR-513a-5p/USP4/TGF-β1 axis.

### Knockdown of LINC01234 Notably Attenuated the Tumor Growth of Liver Cancer *in vivo* Through Inactivation of TGF-β Signaling

**FIGURE 6:**
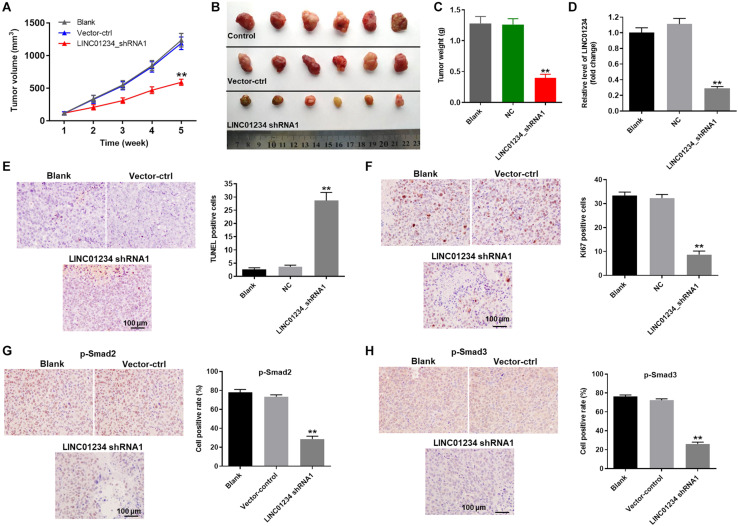
Knockdown of LINC01234 notably inhibited the tumor growth of liver cancer *in vivo* through inactivation of TGF-β signaling. Mice were subcutaneously injected Huh-7 cells transfected with vector-control or LINC01234 shRNA1 after tumors in mice were allowed to grow for 5 weeks. **(A)** Volumes of tumors were measured at the indicated times after transplantation. At the end of study, **(B)** tumor tissues were collected and imaged. **(C)** Tumors of each mouse were weighted. **(D)** The expression of LINC01234 in tumor tissues of mice was detected by qRT-PCR. **(E)** The apoptotic cells in tumor tissues of mice were detected by TUNEL staining. **(F)** Ki67 staining was performed to test the proliferation of liver cancer cells. **(G,H)** The expressions of p-Smad2 and p-Smad3 in tumor tissues of mice were detected by IHC staining. **P < 0.01 compared to control.

Finally, to detect the effect of LINC01234 on liver cancer *in vivo*, xenograft mice model was established. As shown in Figures 6A,B, tumor sizes of mice were significantly decreased by LINC01234 knockdown. Consistently, knockdown of LINC01234 notably downregulated the tumor weights of mice (Figure 6C). Meanwhile, the expression of LINC01234 in tissues of mice was greatly inhibited by LINC01234 knockdown (Figure 6D). Furthermore, Ki67 and Tunnel assays indicated tumor cell proliferation was obviously suppressed by LINC01234 shRNA1 via inducing apoptosis (Figures 6E,F). The expressions of p-Smad2 and p-Smad3 in tumor tissues were notably decreased by LINC01234 knockdown (Figures 6G,H). Altogether, knockdown of LINC01234 notably attenuated the tumor growth of liver cancer through inactivation of TGF-β signaling *in vivo*.

## Discussion

LncRNAs are a group of non-coding RNAs widely distributed in humans (23), which are different from linear non-coding RNAs such as miRNAs. It has been reported that lncRNAs may mediate upregulation or downregulation of gene expression (24), and lncRNAs are stable and widely expressed in many tumor tissues (25, 26). It suggests the possibility that lncRNAs may be involved in paracrine signaling or cell-to-cell crosstalk. The present findings indicate that LINC01234 knockdown could suppress liver cancer cell proliferation via inducing apoptosis. To the best knowledge, this study firstly explored the function of LINC01234 in liver cancer. Current study suggested that LINC01234 likely promote tumorigenesis of liver cancer, particularly during the advanced stages of the disease; LINC01234 might serve as an important biomarker for diagnosis and treatment of liver cancer.

MiRNAs are known to play important roles in the progression of multiple diseases, including liver cancer (17, 27). Our research found miR-513a-5p antagomir partially reversed the inhibitory effect of LINC01234 knockdown on liver cancer, further verified the function of miR-513a-5p in liver cancer progression. In addition, Zhu et al. (28) demonstrated that MRVI1-AS1 enhances nasopharyngeal cancer malignancy by sponging miR-513a-5p. Our present findings are consistent with these results, confirming that miR-513a-5p could act as a key mediator in malignant tumors. However, a previous report indicated that LINC01234 silencing could exert an anti-oncogenic effect in esophageal cancer cells through sponging miR-193a-5p (29). This difference may result from the different tumor type.

It was recently suggested that USP4 plays a key role in multiple malignant tumors (30, 31). In addition, USP4 reportedly mediates cell proliferation, survival and metastasis (32, 33). The present study indicated that USP4 is a direct target of miR-513a-5p. It has been previously confirmed that USP4 could act as a tumor promoter in liver cancer (34). These results further confirm the role of USP4 during the development of liver cancer. Moreover, Jiang et al. (35) indicated that miR-148a dysregulation could discriminate poor prognosis of hepatocellular carcinoma in association with USP4 overexpression. Our result was consistent to this previous study, confirming that LINC01234 could mediate the progression of liver cancer via indirectly targeting USP4.

TGF-β signaling plays a key role in various kinds of diseases, including malignant tumors (36, 37). It has been reported that TGF-β can activate Smad2 and Smad3 (38). Moreover, Smad4 is known to be a core of TGF-β signaling (39). In this study, we found that LINC01234 knockdown downregulated the expression of p-Smad2, p-Smad3 in liver cancer cells and increased the level of Smad4 in Cytoplasm of Huh-7 cells. Based on these data, the mechanism underlying the anti-tumor effects of LINC01234 knockdown was associated with the inactivation of TGF-β signaling pathway. According to Zhang et al. (33), USP4 could positively regulate TGF-β signaling pathway. Current study was consistent to this data, suggesting that LINC01234 could modulate the tumorigenesis of liver cancer via mediation of USP4/TGF-β axis.

Besides, we also found that the expression of E-cadherin, α-SMA and vimentin were notably regulated by LINC01234 shRNA1 in Huh-7 cells. E-cadherin, α-SMA and vimentin played important roles in EMT process (9). Miettinen et al. (40) revealed that activation of TGF-β signaling could enhance the EMT process. Consistent to these findings, we suggested that LINC01234 could promote liver cancer through activation of TGF-β1/EMT signaling.

Components of the PI3K/Akt pathway it is commonly activated in more types of cancer (15). In the current research, LINC01234 knockdown significantly inactivated PI3K/Akt signaling. An earlier report similarly found that inactivation of PI3K/Akt signaling contributes to cancer cell apoptosis (41). Moreover, our findings suggested that knockdown of LINC01234 inactivated EMT process in liver cancer cells. Xiao et al. (42) revealed that USP4 could activate PI3K/Akt to regulate the progression of peritoneal dialysis. Activation of PI3K/Akt could cause the upregulation of EMT process (43). Together with these reports, we suggested that USP4 may promote EMT process in liver cancer through activation of PI3K/Akt. Frankly speaking, the interaction between TGF-β signaling and PI3K/Akt in the condition of LINC01234 knockdown was still unclear and more investigation are needed.

## Conclusion

To sum up, LINC01234 was upregulated in liver cancer. In addition, LINC01234 knockdown could inhibit the growth, migration and invasion of liver cancer cells via mediating the miR-513a-5p/USP4 axis. Thus, LINC01234 may act as a key biomarker for diagnosis and treatment of liver cancer.

## Data Availability Statement

The raw data supporting the conclusions of this article will be made available by the authors, without undue reservation.

## Ethics Statement

The raw data supporting the conclusions of this article will be made available by the authors, without undue reservation.

## Author Contributions

WX, KL, CS, XW, WD, JW, and JL conceived and supervised the study. YL, BX, XL, and JL designed the experiments. All authors reviewed the results and approved the final version of the manuscript.

## Conflict of Interest

The authors declare that the research was conducted in the absence of any commercial or financial relationships that could be construed as a potential conflict of interest.

## Footnotes

1 http://starbase.sysu.edu.cn/

## Supplementary Material

FIGURE S1 Knockdown of LINC01234 significantly induced the apoptosis of HepG2 cells. The apoptotic HepG2 cells were examined by flow cytometry.

FIGURE S2 The melt curve plots of primers.

## Abbreviations

lncRNAs: long non-coding RNAs
USP4: ubiquitin specific protease 4.
