# Corrigendum: Knockdown of lncRNA LINC01234 Suppresses the Tumorigenesis of Liver Cancer *via* Sponging miR-513a-5p

**DOI:** 10.3389/fonc.2020.636847

**Published:** 2021-04-07

**Authors:** Wen Xu, Kesang Li, Changfeng Song, Xiaotong Wang, Yueqi Li, Baixue Xu, Xin Liang, Wanli Deng, Junqing Wang, Jianwen Liu

**Affiliations:** ^1^ State Key Laboratory of Bioreactor Engineering and Shanghai Key Laboratory of New Drug Design, School of Pharmacy, East China University of Science and Technology, Shanghai, China; ^2^ Department of Hematology and Oncology, Hwa Mei Hospital, Ningbo Institute of Life and Health Industry, University of Chinese Academy of Sciences, Ningbo, China; ^3^ Key Laboratory of Diagnosis and Treatment of Digestive System Tumors of Zhejiang Province, Ningbo, China; ^4^ Department of TCM Oncology, Putuo District Central Hospital, Shanghai University of Traditional Chinese Medicine, Shanghai, China; ^5^ Department of Hepatobiliary Surgery, Ruijin Hospital, Shanghai Jiao Tong University School of Medicine, Shanghai, China

**Keywords:** liver cancer, LINC01234, USP4, miR-513a-5p, TGF-β

In the published article, there was an error in affiliation 4. Instead of “Department of Oncology, Shuguang Hospital, Shanghai University of Traditional Chinese Medicine, Shanghai, China”, it should be “Department of TCM Oncology, Putuo District Central Hospital, Shanghai University of Traditional Chinese Medicine, Shanghai, China”.

The authors apologize for this error and state that this does not change the scientific conclusions of the article in any way. The original article has been updated.

