# Supplementary figures and images for: Knockdown of lncRNA LINC01234 Suppresses the Tumorigenesis of Liver Cancer via Sponging miR-513a-5p
Source: Front Oncol. 2020 Oct 16;10:571565. doi: 10.3389/fonc.2020.571565 (PMC7597595; doi:10.3389/fonc.2020.571565)

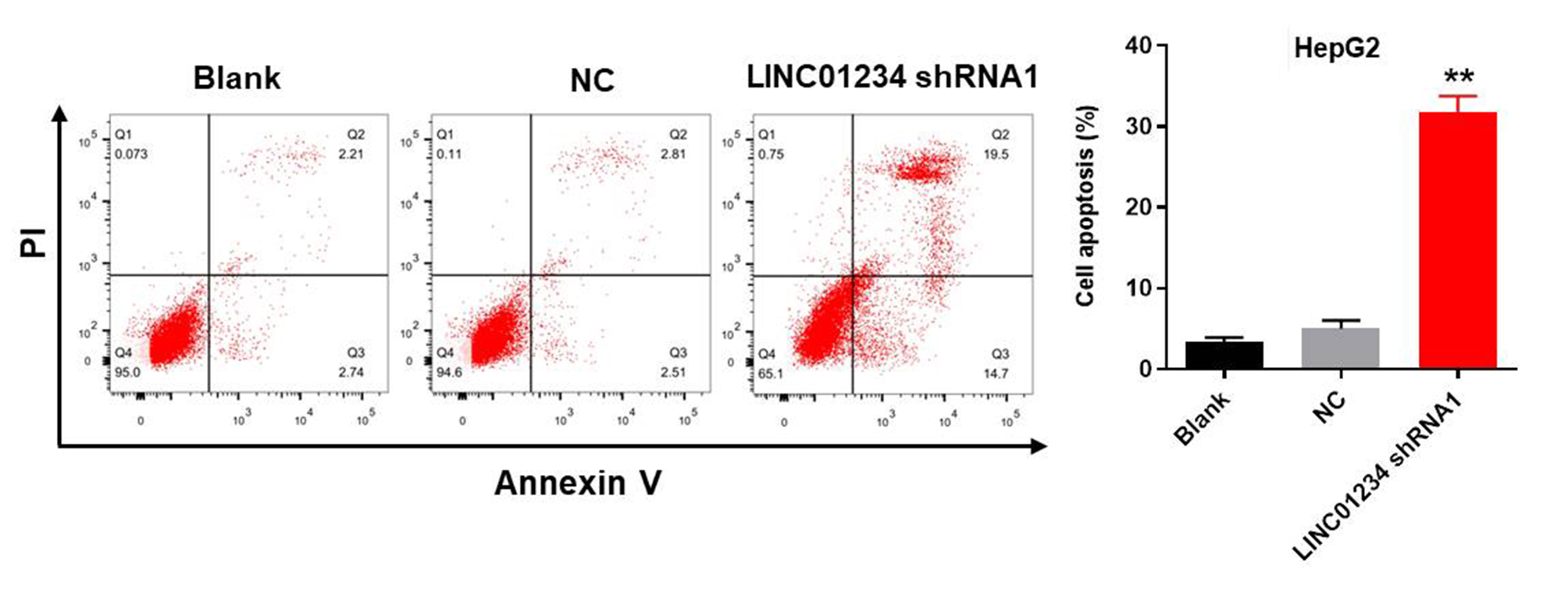

Supplement: FIGURE S1 — Knockdown of LINC01234 significantly induced the apoptosis of HepG2 cells. The apoptotic HepG2 cells were examined by flow cytometry. [file Image_1.JPEG]

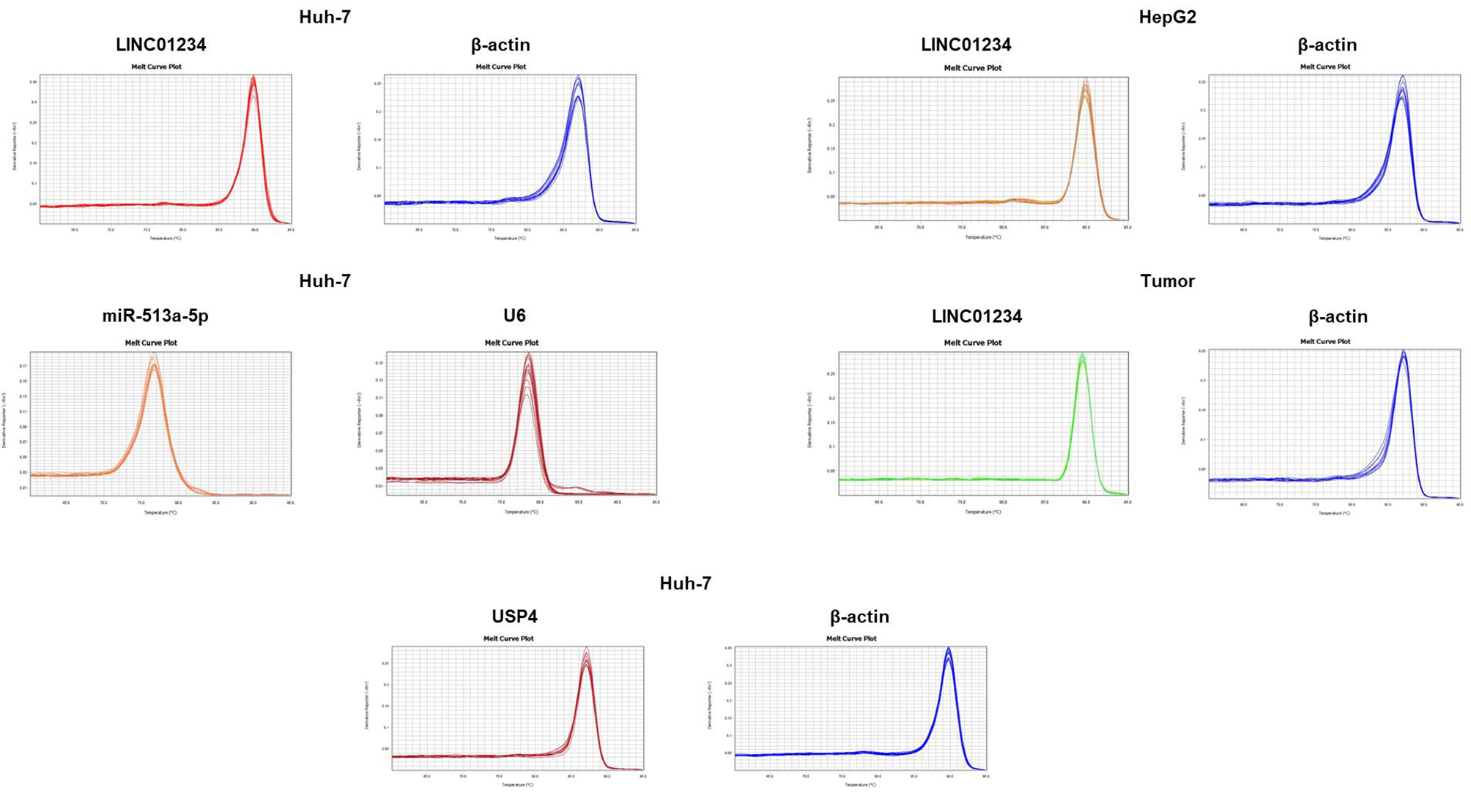

Supplement: FIGURE S2 — The melt curve plots of primers. [file Image_2.JPEG]
